# Chemically deposited palladium nanoparticles on graphene for hydrogen sensor applications

**DOI:** 10.1038/s41598-019-40257-7

**Published:** 2019-03-06

**Authors:** Xiaohui Tang, Pierre-Antoine Haddad, Nathalie Mager, Xin Geng, Nicolas Reckinger, Sophie Hermans, Marc Debliquy, Jean-Pierre Raskin

**Affiliations:** 1 ICTEAM Institute, Université catholique de Louvain (UCL), Place du Levant, 3, 1348 Louvain-la-Neuve, Belgium; 2 IMCN Institute, Université catholique de Louvain (UCL), Place L. Pasteur 1, 1348 Louvain-la-Neuve, Belgium; 3 Materials Science Department, University of Mons, 7000 Mons, Belgium; 4 Department of Physics, University of Namur, Rue de Bruxelles 61, 5000 Namur, Belgium

## Abstract

Graphene decorated by palladium (Pd) nanoparticles has been investigated for hydrogen sensor applications. The density of Pd nanoparticles is critical for the sensor performance. We develop a new chemical method to deposit high-density, small-size and uniformly-distributed Pd nanoparticles on graphene. With this method, Pd precursors are connected to the graphene by π-π bonds without introducing additional defects in the hexagonal carbon lattice. Our method is simple, cheap, and compatible with complementary metal-oxide semiconductor (CMOS) technology. This method is used to fabricate hydrogen sensors on 3-inch silicon wafers. The sensors show high performance at room temperature. Particularly, the sensors present a shorter recovery time under light illumination. The sensing mechanism is explained and discussed. The proposed deposition method facilitates mass fabrication of the graphene sensors and allows integration with CMOS circuits for practical applications.

## Introduction

Hydrogen gas (H_2_) is one of the most promising green energy sources. It is already used in space industry and is considered as a potential candidate to replace engine fuels in automotive and industrial applications due to its high energy density, renewability, and ecofriendly nature^1^. However, the low spark ignition energy (0.02 mJ) and wide flammable range (4–75%) are big concerns for safe production, transportation, and storage^2,3^. For safe applications, it is necessary to develop H_2_ sensors with practical methods for fast and accurate leak detection. Conventional and commercial H_2_ sensors are mainly based on metal oxide materials, such as SnO_2_, TiO_2_, In_2_O_3_ and ZnO^4–7^. Unfortunately, these sensors usually have a low selectivity and operate at high temperatures (between 180 and 500 °C). To overcome these shortcomings, catalytic metal particles, such as palladium (Pd), platinum (Pt), gold (Au), and silver (Ag), are added to decorate metal oxides and improve the sensor performance^8–10^.

It is important to point out that Pd itself is also a sensing material for H_2_. The other metal particles only act as catalysts while sensing is performed by the underlying metal oxides^11^. Pd has a superior H_2_ solubility at room temperature and a good selectivity towards H_2_^12,13^. Furthermore, it has the highest sticking and diffusion coefficients^14^. Therefore, various approaches using Pd as the sole sensing material have also been exploited and investigated in the literature. They can be classified into two groups: Pd-film sensors^15–17^ and Pd-nanostructure sensors^18–21^. The high cost of the Pd-film sensor hinders its practical usage. Moreover, for low H_2_ concentrations (less than 1%), the Pd-film sensor has a long response time^22^. For high H_2_ concentrations (more than 2%), the Pd lattice expansion leads to the Pd film buckle or collapse, making the sensor unstable, irreversible, and unreliable^23^. The Pd-nanostructure sensors include nanowires, nanochains, nanotubes, and nanocomposites. The very low conductance of these discontinuous nanostructures induces a high electrical noise, thereby degrading the sensor response. Although the single Pd nanowire sensors show better performance in sensitivity and power consumption^24,25^, the fabrication of the single Pd nanowire requires electron-beam lithography tools or complex processes^26^.

Recently, graphene has attracted much attention for sensor applications. Its high surface-to-volume ratio makes graphene sensors able to detect a single molecule and its extremely high carrier mobility ensures low electrical noise and energy consumption^27,28^. Particularly, its outstanding structural properties accelerate the diffusion of H atoms^29^. Although pure graphene is chemically inert and has a poor sensitivity to H_2_ detection^30–33^, graphene decorated by Pd nanoparticles (hereafter referred to as ‘Pd-decorated graphene’) has a high response to H_2_. According to a literature survey, we summarize in Table 1 the recently published H_2_ sensors based on Pd-decorated graphene, Pd/graphene composites, and related materials, operating at room temperature.

**Table 1:** Summary of recent research results for H_2_ sensors based on Pd-decorated graphene, Pd/graphene composites, and related materials, operating at room temperature.

Pd Deposition Methods	Sensing Material	Pd NP diameter (d) or Pd layer thickness (t)	Graphene Synthesis Method	Response Value	Response/Recovery Time	Selectivity	Reference
Galvanic displacement	PMMA/Pd NP/CVD-G	d = 20 nm	CVD	66.37% (20000 ppm)	1.81/5.52 min	CO, NO_2_, CH_4_	^70^
e-beam evaporation	Pd NP/MLGN	t = 1 nm	Expanded flake graphite	55% (40 ppm)	21/23 s*	NH_3_, O_2_ cross-sensitivity	^44^
e-beam evaporation	Pd NP/CVD-G	t = 1 nm	CVD	4.1% (500 ppm)	3.5/7.77 min		^45^
Thermal evaporation	Pd NP/CVD-G	t = 3 nm	CVD	32.9% (1000ppm)	10/3.5 min		^32^
Electrochemical deposition	Pd NP/SWNT	d = 35 nm		0.4% (100ppm)**	18/20 min		^71^
e-beam evaporation	Pd NP/G-NRs (200-nm-thick)	t = 2 nm	PECVD	5.8% (1000 ppm)	1/1.5 min (80% recovery)		^13^
Chemical route	Pd-Pt NP/GO	t = 7.6 nm	Hummers method	4.2% (20000 ppm)	2/18 s		^34^
Magnetron sputtering	Pd film/G/Exfoliated G	t = 30 nm	Exfoliated	4.5% (10000 ppm)	1/5 min (36.8%)		^72^
e-beam evaporation	Pd NP/CVD-G Hall bar	t = 1 nm	CVD	2.5% (25 ppm)	10/20 min	CO_2_ O_2_ CH_4_	^73^
Electrochemical depositing	FPNCs/CVD-G	300/80 nm (size/length)	CVD	7% (10 ppm)	50 s	NO_2_, NH_3_	^74^
Chemical route	Pd NP/rGO	d = 10 nm	rGO	7% (1000 ppm)	5/10 min	O_2_ NO_2_ CO CO_2_ N_2_	^35^
**Chemical route**	**Pd NP/CVD-G**	**d = 10 nm**	**CVD**	**5.88% (10000 ppm)**	**3/9 min**	**CH**_**2**_ **O NH**_**3**_	**This work**

PMMA: poly(methyl methacrylate), d: nanoparticle diameter, t: Pd layer thickness, Pd NP: palladium nanoparticles, CVD: chemical vapor deposition, G: graphene, MLGN: multi-layer graphene nanoribbon, SWNT: single-walled carbon nanotubes, NRs: nanorods, rGO: reduced graphene oxide. FPNCs: Flower-like Pd nanoclusters. The rest of the abbreviations can be found in the related references.

*In the related reference, the response/recovery time is defined as the time for 50% of the maximum Δ*R/R* change/recovered.

**The results are obtained from hydrogen-desorption-enhancing air (80% RH).

From Table 1, we note that electron-beam and thermal evaporations are the most common processes for Pd deposition on graphene. However, both processes require complex machines and skilled operators. The vacuum system of the evaporation machines consumes much electrical power and thus the process cost is high. Moreover, the thickness control of the thin Pd layer (determining nanoparticle density, size, and uniform) requires careful measurements. The density of Pd nanoparticles is critical for the sensor performance^34,35^. Specifically, it is observed that the higher the Pd nanoparticle density on graphene (more surface reaction sites), the higher the sensor response.

In this work, we synthesize a Pd precursor solution by a new chemical method. The exact molecular structure of the Pd precursor is confirmed by proton nuclear magnetic resonance spectrometry, thermogravimetric analysis, Fourier Transform Infra-red spectrometry, and high resolution electrospray ionization mass spectrometry. When graphene is immersed in this solution for a few minutes, the Pd molecular precursor adsorbs on the carbon surface by π-π stacking. High-density, small-size and uniform-distributed Pd nanoparticles are obtained by further thermal treatment. This method is simple, cheap, and compatible with complementary metal-oxide semiconductor (CMOS) technology for mass production of the sensors and their integration within CMOS circuits. We characterize the Pd-decorated graphene by transmission electron microscopy, Raman spectroscopy and x-ray photoelectron spectroscopy. The results indicate that the Pd nanoparticles are immobilized onto the graphene without introducing additional defects. Furthermore, the density and size of Pd nanoparticles could be well controlled by tuning the immersing time and/or the concentration of the Pd precursor solution.

Using the Pd-decorated graphene, we fabricate hydrogen sensors on 3-inch silicon wafers. The sensors show high performance at room temperature. The H_2_ detection of the Pd-decorated graphene sensor is mainly based on the conversion of Pd to palladium hydride, which includes 3 stable complexes: PdH, PdH_2_^I^, and PdH_2_^III^. In the following sections, we use PdHx (x = 1 or 2) to stand for 3 stable complexes. In other words, when x = 1, PdH_x_ stands for PdH, while x = 2, PdH_x_ stands for PdH_2_^I^ and PdH_2_^III^ since PdH_2_^I^, and PdH_2_^III^ nearly have the same binding energies (see the following). The sensor recovery is closely related to the re-conversion of PdH_x_ to Pd, which is a slow chemical process. As a consequence, the signal recovery of the sensors is slow and incomplete at room temperature. In our previous works, heating was used to help the signal recovery^36,37^. Heating requires a resistance heater, which could trigger an explosive reaction, increasing the danger of sensor operation and degrading the sensor itself. In the present work, we replace heating by illumination (light-emitting diode, LED), to shorten the recovery time. Ultra-violet (UV) light is a useful light source for dissociating surface adsorbed species, which allows gas sensing at room temperature^38,39^. However, UV sources are expensive and power hungry. Moreover UV sources damage eyes and plastic substrates of the sensors. We chose a visible light source (purple light) to improve sensor performance because it is inexpensive, energy saving and environmentally friendly, compared to UV sources.

## Results

### Characterization of the Pd precursor solution

The Pd precursor solution is a Pd(bipyridine)(pyrene)_2_ complex. Its synthesis procedure can be found in the method section. The molecular structure of Pd(bipyridine)(pyrene)_2_ is determined using a 300 MHz Brucker NMR spectrometer with CD_2_Cl_2_ as solvent. Proton nuclear magnetic resonance (^1^H-NMR) spectra are recorded at 20 °C. The analysis results are summarized as follows: ^1^H NMR (300 MHz, CD_2_Cl_2_) δ 2.11 (q, 4 H, J = 8 Hz), 2.49 (t, 4 H, J = 7 Hz), 3.27 (t, 4 H, J = 8 Hz), 7.47 (m, 2 H), 7.77 (m, 2 H), 7.91–8.12 (m, 18 H), 9.28–9.32 (m, 4 H) ppm.

The Pd residues in Pd(bipyridine)(pyrene)_2_ are examined by a thermogravimetric analysis system (TGA-DSC SDT 2960 from TA Instruments). A sample of about 2 mg is placed in an alumina container with a volume of 70 μl. The examination is carried out under a 100 ml/min nitrogen flow with a heating rate of 10 °C/min. When the Pd(bipyridine)(pyrene)_2_ sample is heated up to 400 °C, the Pd residues are 26%, while the mass loss is 74%. According to the chemical formula, the Pd residues and the mass loss are calculated to be 12.7 and 87.3%, respectively. The difference arises from the incomplete reduction of Pd molecules and/or combustion of ligands in the nitrogen atmosphere due to the absence of active reducing agent, such as hydrogen.

The Pd ligation is characterized by a Brucker Fourier Transform Infra-Red (FTIR) spectrometer (type EQUINOX 55). The sample is prepared by grinding Pd(bipyridine)(pyrene)_2_ (1 wt. %) with potassium bromide (KBr) powder and applying pressure to obtain a transparent pellet. The infrared spectra of Pd(bipyridine)(pyrene)_2_ are recorded between 4000 and 400 cm^−1^ with a resolution of 4 cm^−1^. The presence of the ligands in the complex is confirmed by peaks at 1371 and 1603 cm^−1^, corresponding to symmetric and asymmetric COO^−^ stretching modes, respectively.

The mass of the [Pd(bipyridine)(pyrene)_1_]^+^ ion is measured by high-resolution electrospray ionization mass spectrometer (HRMS (ESI)). The sample is ionized by a direct introduction through negative mode under the following conditions: capillary temperature of 320 °C, spray voltage of 3.5 kV and sheath gas flow rate of 5 (in arbitrary units), corresponding to about 1.5 L/min. HRMS results are recorded using Q-Exactive orbitrap (from ThermoFisher). The result indicates that the positive ion [Pd(bipyridine)(pyrene)_1_]^+^ has a mass of 549.07998 *m/z*, which is very close to the calculated value of 549.07889 *m/z*, confirming its chemical nature.

### Observation of Pd-decorated graphene by transmission electron microscopy (TEM)

**Figure 1:**
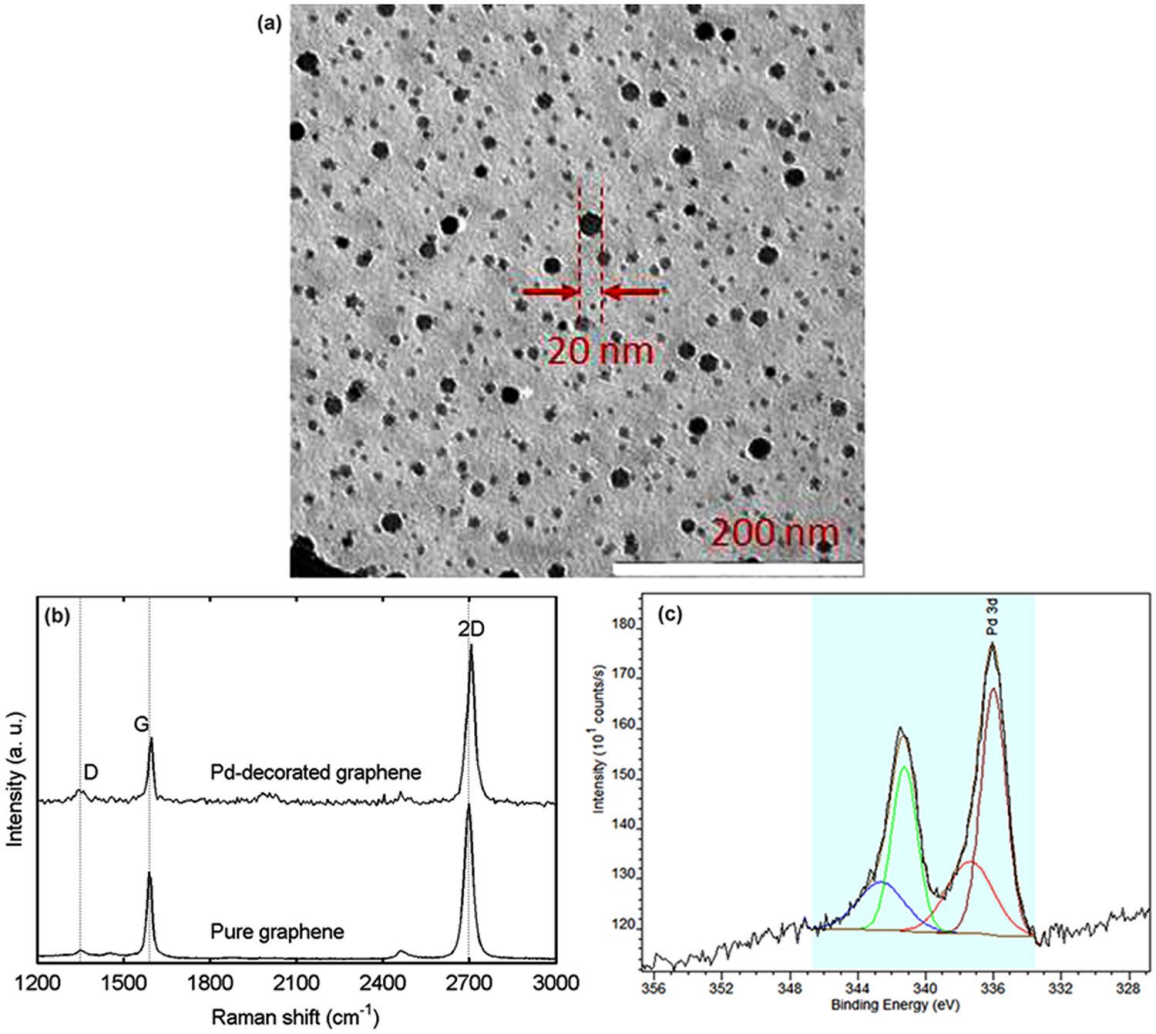
Physical characteristics: (**a**) transmission electron microscopy image, Pd nanoparticles having an average diameter of 10 nm, (**b**) Raman spectra for pure graphene (bottom) and Pd-decorated graphene (top), and (**c**) X-ray photoelectron spectrum of Pd-decorated graphene: Pd_3d_ doublet for Pd^0^ brown & green, Pd_3d_ doublet for Pd^II^ red & blue.

In order to determine the density and size of Pd nanoparticles, a reference sample is prepared: the CVD-grown graphene is transferred on a TEM grid using the method in^40^. The TEM grid covered with graphene is subjected to the same graphene transfer and Pd-decoration procedures as described in the method section. Figure 1a shows a TEM image of the reference sample. This image is obtained by a LEO 922 Omega Energy Filter TEM, operating at 200 kV. The largest diameter of observed Pd nanoparticles is 20 nm. The average diameter of the Pd nanoparticles is 10 nm, statistically calculated using an image analysis software (‘Image J’^41^) based on the TEM image with a size of 450 × 450 nm^2^. We can assume that the TEM image shows the real morphology of the Pd-decorated graphene sensor, since the reference sample and the sensor endure a strictly identical procedure. Moreover, the graphene in the reference sample and the sensor, characterized by Raman spectroscopy, have similar quality. The density and size of Pd nanoparticles on graphene could be tailored by controlling the immersing time and/or the concentration of the Pd precursor solution.

### Evaluation of Pd-decorated graphene by Raman spectroscopy

A LabRam Horiba spectrometer is employed to evaluate the Pd-decorated graphene on the SiO_2_/substrate. Figure 1b displays a typical Raman spectrum for the Pd-decorated graphene. The Raman spectrum of pure graphene without Pd decoration is also shown at the bottom of the figure for reference. For pure graphene, the G and 2D peaks are observed at around 1590 and 2687 cm^−1^, respectively. The I_2D_/I_G_ peak ratio is 1.8 and the full width at half maximum (FWHM) of the 2D peak is 35 cm^−1^. Moreover, the defect-activated D peak, located at 1356 cm^−1^, is very weak. These observations clearly verify that the pure graphene is single layer with few structural defects. The statistical data from many measured points show that after Pd decoration, the G and 2D peaks are only slightly upshifted, the I_2D_/I_G_ peak ratio remains unchanged, and the FWHM of the 2D peak is not broadened. Importantly, the intensity of the D peak is still low. The slight upshift of the G and 2D peaks is most probably due to strain originating from Pd atom incorporation or doping. However, the graphene surface has no significant deformation. The low intensity of the D peak indicates that the symmetry of the graphene lattice is not broken by Pd atom incorporation^42^. Moreover, the D’ peak (at 1620 cm^−1^), corresponding to sp^3^ bonding, does not appear at the right shoulder of the G peak^43^. These facts confirm that our chemical method for Pd decoration does not damage the graphene structure and hence proves that the Pd precursors are connected with the graphene by π-π bonds via the pyrene moiety without introducing additional defects.

### Analysis of Pd-decorated graphene by X-ray photoelectron spectroscopy (XPS)

The oxidation state of the metal in Pd-decorated graphene is analyzed by XPS (using a SSI-X-probe (SSX-100/206) Fisons spectrometer). The spectra are recorded at constant pass energy of 150 eV. The Pd-decorated graphene sensor is directly stuck onto an insulating ceramic sample holder (Macor, Switzerland) by double-face adhesive tape. A nickel grid is fixed 2 mm above the sensor. An electron flood gun is set at 8 eV to overcompensate the positive charging of the sensor during the analyses. The analyzed area is approximately 1.4 mm^2^. The XPS results are fitted with the CasaXPS software using a sum of Gaussian/Lorentzian (85/15) after subtraction of a Shirley-type baseline.

The Pd_3d_ core level spectrum is displayed in Fig. 1c. The Pd_3d_ peak is a doublet comprising the Pd_3d5/2_ and Pd_3d3/2_ peaks. The signal is decomposed into a sum of two doublets corresponding to Pd^0^ and Pd^II^. The XPS result reveals two Pd states: the reduced metal Pd (Pd^0^) in nanoparticles and the oxidized Pd (Pd^II^) in complex molecules or surface oxides. The 0.447 at.% surface percentage of Pd_3d_ is decomposed into 0.292 at.% Pd^0^ and 0.155 at.% Pd^II^. It should be noted however that the Pd^0^ peak should present a higher asymmetry (not taken into account here), hence it is always underestimated compared with the Pd^II^ component. A small amount of oxidized Pd^II^ is found due to incomplete reduction or surface re-oxidation as the sample is handled in air. Previously, we clearly identified the presence of the Pd nanoparticles by TEM imaging. Here, the XPS measurements quantitatively provide the surface percentage of reduced Pd atoms in nanoparticles. Conclusively, the present chemical method succeeds in decorating graphene mainly with reduced Pd nanoparticles.

### Performance of Pd-decorated graphene sensors for H_2_ detection

**Figure 2:**
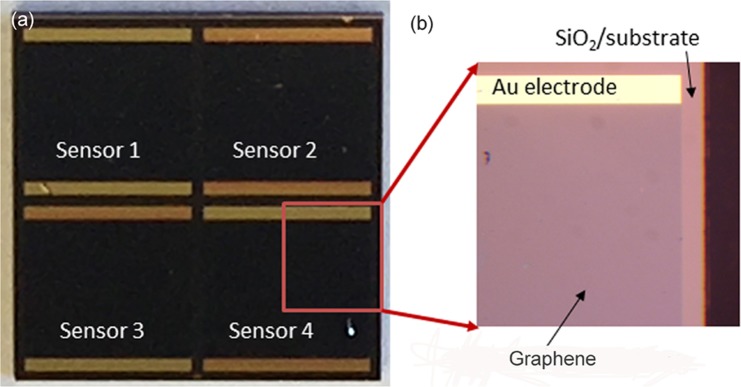
Optical microscope images: (**a**) a chip made of 4 Pd-decorated graphene sensors and (**b**) zoom in a corner of sensor # 4.

**Figure 3:**
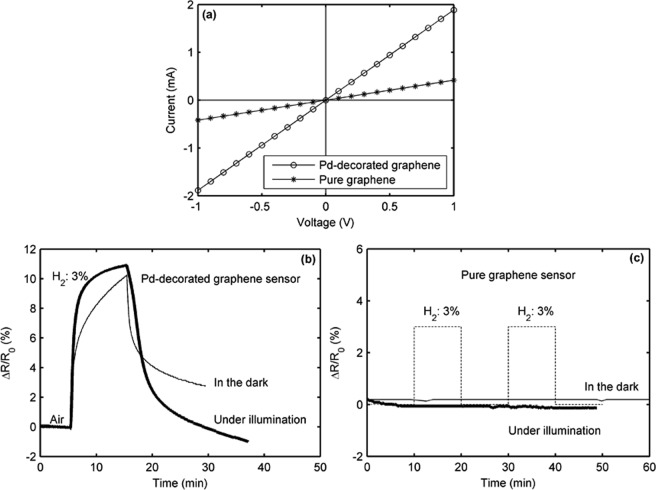
Electrical behavior: (**a**) current-voltage characteristics of a graphene sensor before and after Pd decoration, (**b**) resistance responses of a Pd-decorated graphene sensor for 3% H_2_ under purple light illumination (thick line) and in the dark (thin line), and (**c**) resistance responses of a pure graphene sensor for 3% H_2_ at 20 °C in air.

Figure 2a is a top optical image of a fabricated dice, which includes 4 Pd-decorated sensors. Figure 2b zooms in on the corner of sensor #4, in which the graphene layer on SiO_2_/Si can be clearly seen. Figure 3a shows the current-voltage curves of a graphene sensor before and after Pd decoration. The sensor resistance has ohmic characteristics, indicating that the contact resistance of the Au electrodes is quite low. It can be seen that the resistance of the sensor is significantly decreased after Pd decoration. The reason is that the graphene is doped by Pd (Raman results confirm it). This will be explained in detail in the discussion section. Figure 3b demonstrates the resistance responses of a typical Pd-decorated graphene sensor for 3% H_2_ at 20 °C. When the sensor is exposed to H_2_, either under purple light illumination (thick curve) or in the dark (thin curve), its resistance is increased. However, the sensor recovery is extremely slow and even incomplete in the dark. Under purple light illumination, the sensor performance is obviously improved, especially the recovery characteristic. For comparison, the resistance response of a pure graphene sensor without Pd decoration for 3% H_2_ is also provided in Fig. 3c. Only a negligible response is observed under illumination and in the dark. This is due to the fact that pure graphene is chemically inert and weakly binding with H_2_ molecules^44–46^. This result indicates that Pd nanoparticles play a catalytic role in graphene doping by H_2_ molecules^47^.

**Figure 4:**
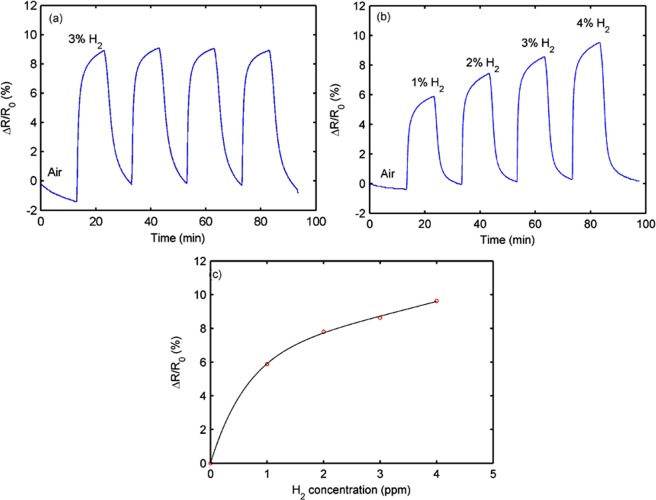
Resistance responses of Pd-decorated graphene sensor at 20 °C in air: (**a**) exposed to 4 cycles of 3% H_2_, (**b**) exposed to different H_2_ concentrations of 1, 2, 3, and 4%, and (**c**) relationship between the resistance response and the H_2_ concentration.

Figure 4a shows the resistance responses of the Pd-decorated graphene sensor under purple light illumination, exposed to 4 cycles of 3% H_2_ at 20 °C. The H_2_ exposure time is 10 min, followed by 10 min of purge with air (50% RH). The sensor has a repeatable response, suggesting good repeatability. Figure 4b shows the sensor responses for different concentrations of H_2_ (1, 2, 3, and 4%). The dependence between the sensor responses and the H_2_ concentrations is given in Fig. 4c, in which the open circles are experimental data and the solid line is the fitting curve with a three-term exponential function given by y = *a*e^*b*x^ + *c*e^*d*x^ + e. For 1% H_2_, a sensor response of 5.88% is achieved. The response and recovery times of the sensor are 3 and 9 min, respectively. These key parameters are listed in Table 1 for comparison purpose. In this work, the investigated concentrations are lower than the flammable limit of H_2_ (4%).

**Figure 5:**
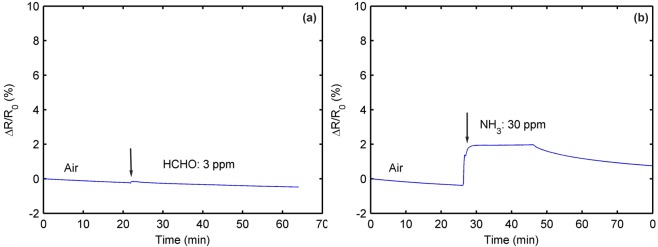
Resistance responses of a Pd-decorated graphene sensor: (**a**) for 3-ppm formaldehyde and (**b**) for 30-ppm ammonia.

From Table 1, we can see that compounds, including C, H, O and N elements, are usually used as interference gases to test the selectivity of the Pd-based sensors. Therefore, we choose formaldehyde (CH_2_O) and ammonia (NH_3_) as interference gases to investigate our sensor. The concentrations of the interference gases are chosen to be higher than the tolerable concentrations in the human body. Specifically, the World Health Organization (WHO) set a 30-min threshold limit of 0.08 ppm for CH_2_O^48^. Some countries established an 8-h permissible exposure limit of 25 ppm for NH_3_^49^. Figure 5a shows the present sensor response exposed to 3 ppm CH_2_O. No sensor response is observed. When the sensor is exposed to 30 ppm of NH_3_, it only produces a slight response of 2% (see Fig. 5b). These results confirm that the present sensor is not influenced by CH_2_O and NH_3_ at the maximum concentrations in normal air.

## Discussion

**Figure 6:**
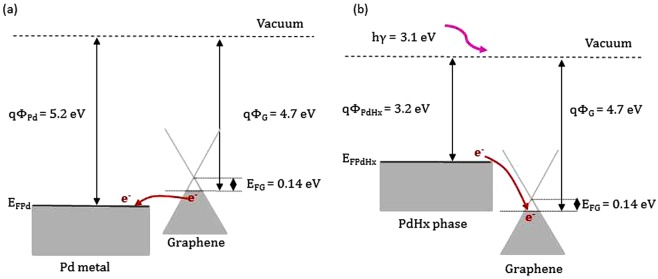
Energy band diagrams (**a**) for Pd and *p*-type graphene and (**b**) for PdHx and *p*-type graphene.

In this study, Pd nano particles are chosen due to its superior hydrogen solubility at room temperature^44,50^. The reaction between H_2_ and Pd leads to two phases^51^. One is α phase (solid solution) and the other is β phase (palladium hydride, PdHx). In the α phase, the interstitial H atoms generated by the dissociation of adsorbed H_2_ diffuse to the Pd nano particles. When the H atom concentrations are higher than a given concentration limit (depending on the temperature and H_2_ pressure), the β phase is formed (the phase diagram can be found in^52^). At room temperature, the saturation of the α phase is reached for hydrogen pressure below 0.01 bar. In this case, the ratio of H/Pd is about 10%. The H_2_ detection mechanism of the Pd-decorated graphene sensor is based on the graphene resistance change, which is mainly induced by the work function change of Pd^13,53,54^. As shown in Fig. 6a, the work functions of Pd and graphene are 5.2^55^ and 4.7 eV^56^, respectively. In general, pure CVD graphene transferred on SiO2/Si substrates behaves like a *p*-type semimetal^57^. The graphene resistance is decreased after the Pd decoration since the work function of Pd is larger than that of graphene^58^. This is beneficial towards the electron transfer from graphene to Pd, enhancing hole density in *p*-type graphene. Our experimental results confirm this fact^59^. The baseline resistance of the pure graphene sensor is decreased from 2395 to 530 Ω after Pd decoration (as shown in Fig. 3a). When the Pd-decorated graphene is exposed to H_2_ at room temperature, H_2_ is dissociated on the Pd surface into H atoms to form PdHx. As shown in Fig. 6b, the work function of PdHx (3.2 eV)^60^ is smaller than that of graphene. In this case, the graphene resistance is increased due to the electron transfer from PdHx to graphene, reducing hole density in *p*-type graphene. The Pd nanoparticles act as reaction sites for H_2_ chemisorption, while graphene is equivalent to an electron reservoir and pathway. The above discussion is suitable for the case in the dark.

We then discuss, referring to Fig. 3b, why the sensor behavior becomes better under illumination. In other words, the sensor response is a shark-fin curve in the dark, while the sensor response closes to a square curve under illumination. During adsorption, the illumination may accelerate the dissociation of H_2_ molecules on the Pd surface, which is the prior step for the formation of the PdHx. The illumination case is similar to the case in ref.^36^, where heating was used to accelerate the dissociation of H_2_ molecules. Both cases are based on the fact that external environment provides additional energies (light and heating energies). This is the reason why the time for reaching the response saturation is shorter under illumination than in the dark. The sensor recovery is closely related to two processes: the re-conversion of PdHx to Pd and the spillover of the H atoms. The former is a slow process at room temperature. The photon energy of purple light is 3.1 eV (purple light wavelength = 400 nm), while the largest binding energy of PdHx is 2.96 eV^36^. As a consequence, the photon energy is large enough to help the PdH_x_ decomposition and release the H atoms, leading to a quicker sensor recovery under illumination. Authors in ref.^61^ also observed a faster recovery under UV light. On the other hand, the photon energy is also sufficient to directly excite the electron jump from the valence band to the conduction band in graphene. Because the Fermi level of the *p*-type graphene is about 0.14 eV (*E*_F_ = *ħv*_F_(*πn*)^1/2^, *ħ* the reduced Planck’s constant, *v*_F_ = 10^6^ ms^−1^ the Fermi velocity in graphene, *n* = 1.5 × 10^12^ cm^−2^ the doping concentration of *p*-type graphene)^62,63^. The electrons, transferred from the PdH_x_ and jumped in the conduction band, provoke an increase of the graphene resistance. This synergistic effect makes the present sensor have a higher response under illumination than in the dark.

## Conclusion

Palladium (Pd) nanoparticles are deposited on graphene by a new chemical method, in which Pd(bipyridine)(pyrene)_2_ complex is synthesized as the Pd precursor solution. In 10-min immersing time, Pd precursors are connected with the graphene by π-π bonds without causing additional defects in the hexagonal carbon lattice and a subsequent thermal treatment forms the desired nanoparticles. Our method is simple, cheap, and compatible with complementary metal-oxide-semiconductor (CMOS) technology. With graphene decorated by Pd nanoparticles, we fabricate hydrogen sensors at the wafer scale with high-throughput. The sensor has a response of 5.88% for 1% H_2_ at room temperature under purple light illumination. The sensor response, the response time and more particularly the recovery time are significantly improved under illumination. The present sensor is not influenced by certain redox gases, such as formaldehyde and ammonia. Our sensors could be integrated with CMOS circuits for developing a multiple sensor platform in the future.

## Methods

### Synthesis of Pd precursor solution

**Figure 7:**
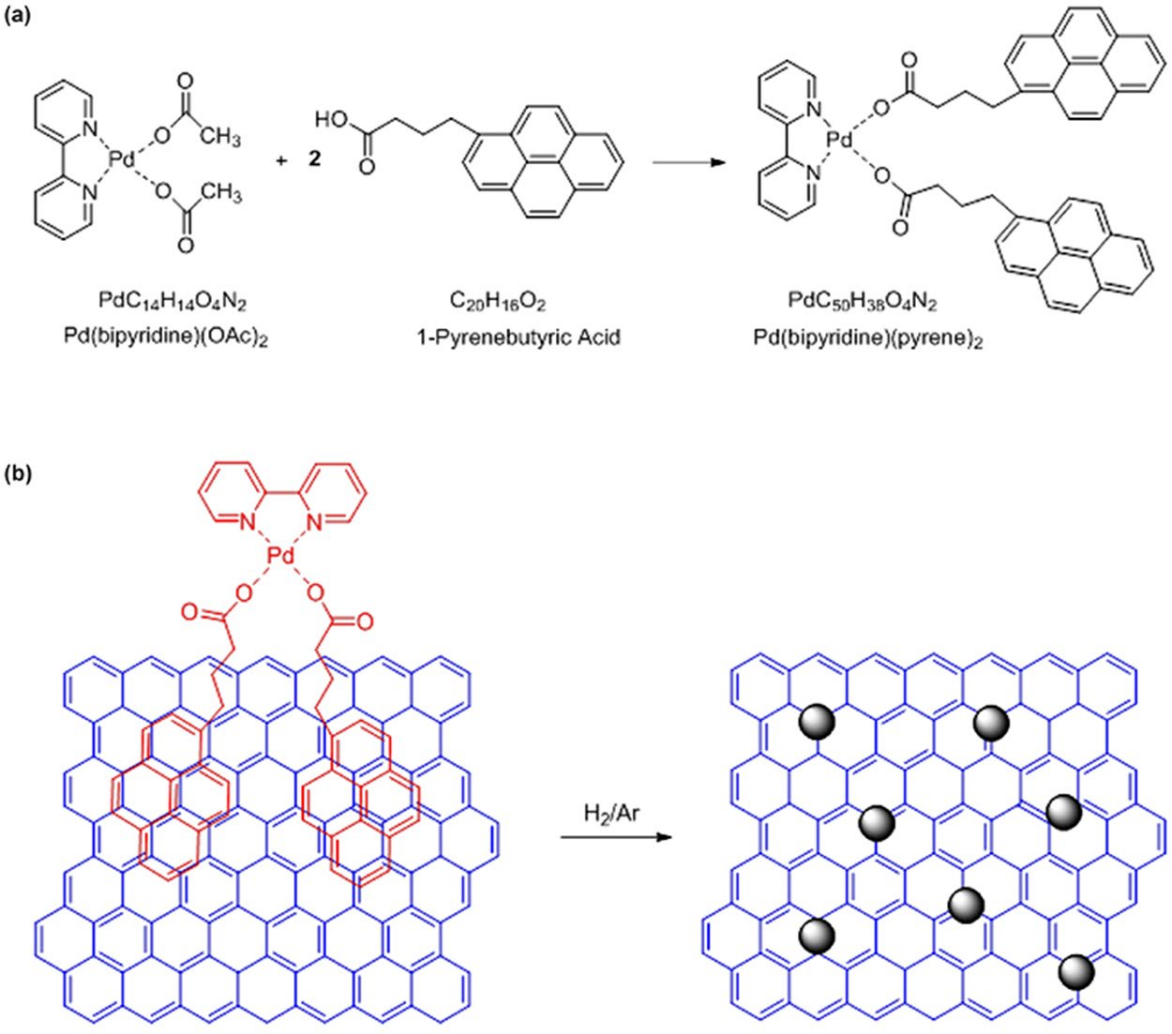
Chemical scheme illustrations: (**a**) Pd precursor (Pd(bipyridine)(pyrene)_2_) synthesis and (**b**) Pd complex molecules (Pd^II^) reduction into nanoparticles (Pd^0^) on graphene.

Figure 7a depicts a chemical scheme illustration for the Pd(bipyridine)(pyrene)_2_ synthesis. 0.055 g Pd(bipyridine)(OAc)_2_ (0.13 mmol, 1 equiv.) and 0.042 g 1-pyrenebutyric acid (0.13 mmol, 1 equiv.) [Alfa Aesar, 97%] are mixed with 15 ml of methanol [VWR, 100%] in a Schlenk flask under argon. The yellow-brown mixture solution is agitated for 5 hours and concentrated to 10 ml in vacuum. The powder is filtered out on a filter paper under air and washed with hexane. The dry yellow powder is transferred in a 3-neck Schlenk equipped with a filter, and recrystallized in 30 ml of acetonitrile [VWR, ≥99.9%]: the mixture is heated to 63 °C and filtered out, and the filtrate is stored under argon overnight. Bright yellow crystals of Pd(bipyridine)(pyrene)_2_ (0.019 g, 0.034 mmol, 24% yield) are recovered, washed with acetonitrile, dried in vacuum, and stored under argon in the dark. The Pd precursor solution is prepared by dissolving 0.0004 g of Pd(bipyridine)(pyrene)_2_ in 10 ml of dichloromethane. It is worth noting that the starting compound Pd(bipyridine)(OAc)_2_ (yield 83%) is easily prepared from palladium acetate [Rocc, Pd 47.27%] and 2,2′-bipyridine [Acros, 99+%] in acetone [VWR, 99.9%] following the literature procedures^64,65^. IR (KBr disks): ν_as_ (COO) 1873, ν_s_ (COO) 1315 cm^−1^. In the synthesis procedure of the Pd precursor solution, all solvents without description are HPLC grade.

### Fabrication of Pd-decorated graphene sensors

The fabrication process of the Pd-decorated graphene sensors is compatible with CMOS technology and involves 4 main steps: (i) preparation of the sensor substrates, (ii) graphene transfer; (iii) definition of the sensing areas and electrodes, and (iv) Pd-nanoparticle decoration. A 3-inch Si wafer (*p*-type, with a resistivity of 10–25 Ω.cm) is used as a starting substrate. A 90-nm-thick SiO_2_ layer is thermally grown on top of the substrate. The SiO_2_ layer acts as an electrical insulator for the sensors and it also allows to observe graphene with a conventional white light microscope^66^. A back gate is built on the backside of the Si wafer by depositing a 200-nm-thick aluminum film. The commercially CVD-grown graphene (from Graphenea Inc.) is transferred to the substrate surface using a supporting layer of poly(methyl methacrylate) (PMMA). The details of the graphene transfer can be found in ref.^67^. The presence of the monolayer graphene is confirmed by Raman spectroscopy measurements. The sensing areas and electrodes are defined on the surface of the graphene/SiO_2_/substrate by a two-step optical lithography. The graphene sensing areas are protected by the photoresist (AZ5214E) and the exposed graphene is etched using inductively-coupled plasma at 30 W RF and 75 W DC for 20 s at 20 mTorr with 50 sccm of O_2_. The residual photoresist is stripped away with acetone. After a second lithography, Au/Ti electrode pairs (95/5 nm) are deposited by electron-beam evaporation and subsequent lift-off process. The thin Ti layer between Au and graphene is used for improving the adhesion of the Au electrodes. In order to reduce the contact resistance, Au/Ti electrodes must be contacted with the graphene side^68^. Finally, the substrate with graphene and electrodes is immersed in the Pd precursor solution for 10 min. This step allows the Pd precursor molecules to establish π-π bonds with graphene. The substrate is carefully washed by a clean solution of dichloromethane for 10 seconds. This rapid cleaning procedure keeps the strong interaction between the Pd precursor molecules and graphene, and removes any other impurities, including the PMMA from graphene transfer and the photoresist residues from the optical lithography steps. As illustrated in Fig. 7b, to reduce the Pd precursor molecules (Pd^II^) into atoms (Pd°) the substrate is annealed in a furnace at 300 °C for 40 min in a forming gas of H_2_/Ar (10/90). The annealing temperature and time could be further reduced. It is emphasized that the Pd-nanoparticle decoration is a post process, which can be integrated with the fabrication of CMOS circuits. The substrate is then cut into many dies with four sensors. Each sensor has a sensing area of 2.7 × 2.3 mm^2^. Preliminary electrical measurements suggest that the commercial graphene is highly *p*-doped since the Dirac point does not appear in the resistance curve when a back-gate voltage is swept from −40 to 40 V.

### Measurement setup

We teste the fabricated sensors with a homemade measurement setup^69^. A purple-light bulb made of 20 LEDs is installed in a sealed plastic chamber, just in front of the tested sensor. The distance between the bulb and the sensor is fixed at 45 mm. The wavelength of purple light is 400 nm. The intensity of purple light is 0.98 mW/cm^2^, measured by means of a photodiode (Thorlabs, FDS100). The power consumption of visible lights is in the order of mW magnitude. It is worth noting that the bulb does not heat the sensor since the temperature of the sensor surface is not significantly increased. The sensor is placed in the middle of the chamber to maintain a constant gas flow for a stable gas reaction. Every measurement is performed under air with 50% relative humidity (RH) and at 20 °C. The level of the relative humidity is controlled by mixing dry and wet air (by bubbling in deionized water at 20 °C). At first, air is supplied to the chamber at 1000 sccm for a certain time to have a stable resistance and rule out any other reactive gas molecules. All the gas flow rates in this work are controlled by mass flowrate controls (MFC, Bronkhorst, high-Tech, Netherlands). Then the sensors are exposed to purple light with an intensity of 0.98 mW/cm^2^ and subsequently H_2_ is introduced with various concentrations (1, 2, 3 and 4%) into the chamber. The present research mainly focuses on around the flammable limit (4%) for fire prevention applications. The relative change in resistance between the two electrodes is used to define the sensor response ∆R/R_0_ = (R_S_ − R_0_)/R_0_ (%), where R_0_ and R_S_ are the sensor resistance before and after exposure to the target gas, respectively. The response time is defined as the time to reach 90% of the total measured resistance change, while the recovery time refers to the time required for recovering the measured resistance to 90% of its original value. The sensor resistance is measured using a semiconductor parameter analyzer inside the chamber by recording the current at a constant voltage of 0.7 V. The back gate of the wafer is connected to the electrical ground during the experiments.
